# Syphilis, Tuberculosis, and Fresh Air

**Published:** 1903-03-07

**Authors:** 


### SYPHILIS, TUBERCULOSIS, AND FRESH AIR.

There is abundant evidence of the inestimable value of "open-air" treatment, not only in tuberculous diseases, but also in a large and increasing number of other affections. Dr. Douty[1] calls attention to its value in early syphilis. He points out that the good effect of pure air and sunlight in phthisis is not due to a local influence on the lungs so much as to an improvement in the blood and all the tissues of the body. Fresh air heightens metabolism and increases resistance to poisons carried by the circulating blood. The increased appetite and power of assimilation, and the graduated exercise act in the same way. All these concomitants of the open-air treatment are important in enabling the individual, with the help of small doses of mercury maintained for several years, to escape the eventual horrors of syphilis. Dr. Douty does not look upon mercury as a "specific" for the disease, though he lays great stress on the value of prolonged administration of small doses of the drug. He has been much impressed by the different effects shown when syphilis takes hold of the various types of undergraduates; the poor, under-fed scholar, if infected, is, as a rule, severely attacked, while the well-to-do athletic undergraduate generally suffers lightly. The latter leads an open-air life, is well fed, and therefore has a high power of resistance. Dr. Douty has come to the conclusion that 30 per cent. of the men patients under his care suffering from phthisis are syphilitics; and he quotes Dr. Klause, of Berlin, as saying that when he was in charge of the venereal department at the Charité at Berlin he noticed that more than 50 per cent. of the patients showed physical signs of lung disease. In drawing his conclusions as to the benefits of open-air treatment for syphilitics it must be presumed, although he makes no mention of it, that Dr. Douty has excluded the cases where patients had phthisis in addition to the syphilis. In concluding he has some very strong opinions to express on the correlation of syphilis and tuberculosis; he goes so far as to say that syphilis is such a potent predisposing cause of tuberculosis, that there is little hope of ever stamping out the latter unless a simultaneous endeavour is made to stamp out syphilis.

[1] Brit. Med. Jour., Feb. 28.

